# 2650. Characteristics of In-Hospital Care and Immediate Post-Discharge Care Status among Older Adults Hospitalized with Respiratory Syncytial Virus Infection, Seasonal Influenza, Acute Myocardial Infarction, and Stroke in the US

**DOI:** 10.1093/ofid/ofad500.2261

**Published:** 2023-11-27

**Authors:** Reiko Sato, Jennifer Judy, Kari Yacisin, Elizabeth Begier, Poorva Sardana, Neha Agrawal, Anchita Goswami, Manvi Sharma

**Affiliations:** Pfizer, Inc., Collegeville, Pennsylvania; Pfizer, New York, New York; Pfizer Inc., Collegeville, Pennsylvania; Pfizer Vaccines, Dublin, Dublin, Ireland; Complete HEOR Solutions (CHEORS), Chalfont, PA, USA, Chalfont, Pennsylvania; Complete HEOR Solutions (CHEORS), Chalfont, PA, USA, Chalfont, Pennsylvania; Complete HEOR Solutions (CHEORS), Chalfont, PA, USA, Chalfont, Pennsylvania; Complete HEOR Solutions (CHEORS), Chalfont, PA, USA, Chalfont, Pennsylvania

## Abstract

**Background:**

Despite increased recognition of the morbidity, mortality, and economic burden of respiratory syncytial virus (RSV) in adults, few studies have documented the status of post-discharge care after RSV hospitalization. This study assessed in-hospital and immediate post-discharge care status in older adults hospitalized with RSV in the US and compared it with other serious medical conditions (influenza, acute myocardial infarction (AMI), and stroke) that have long-standing disease prevention efforts.

**Methods:**

This retrospective cohort study used data from Premier Healthcare Database between January 1, 2015, to December 31, 2019. Adults aged ≥ 65 years hospitalized due to primary diagnosis of RSV, influenza, AMI, or stroke were included. Discharge statuses were used to categorize the levels of immediate post-discharge care as shown in **Figure 1**. Descriptive statistics and Sankey diagrams were used to summarize the four cohorts.

**Figure:**
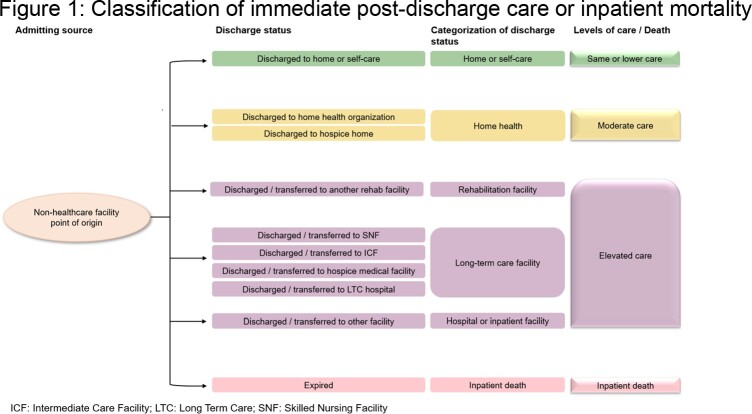

**Results:**

Overall, 2,559 RSV, 174,826 influenza, 208,448 AMI, and 274,102 stroke hospitalizations were identified with similar mean patient ages of 79.8, 76.8, 76.3, and 78.0 years, respectively. Almost all patients ( > 90%) had underlying comorbidities. The mean inpatient length of stay (LOS) was similar in the four cohorts (4.0, 4.4, 3.7, and 4.1 days, respectively), but the prevalence of mechanical ventilator use varied, i.e., 0.4%, 13.7%, 5.9%, and 1.4%, respectively. Intensive Care Unit (ICU) admissions during RSV hospitalizations were infrequent (3.0%) as compared to influenza (16.8%), AMI (24.5%), and stroke (20.5%). The mean ICU LOS for these four cohorts were 2.3, 2.8, 1.7, and 1.7 days in the same order. Immediately following discharge, moderate care was required for 23.9%, 22.9%, 14.1%, and 17.5% of RSV, influenza, AMI, and stroke hospitalizations, respectively. Elevated care was considerably more frequent for stroke hospitalizations (48.1%) than RSV (18.8%), influenza (21.4%), and AMI (19.3%) hospitalizations (**Figure 2**).

**Figure:**
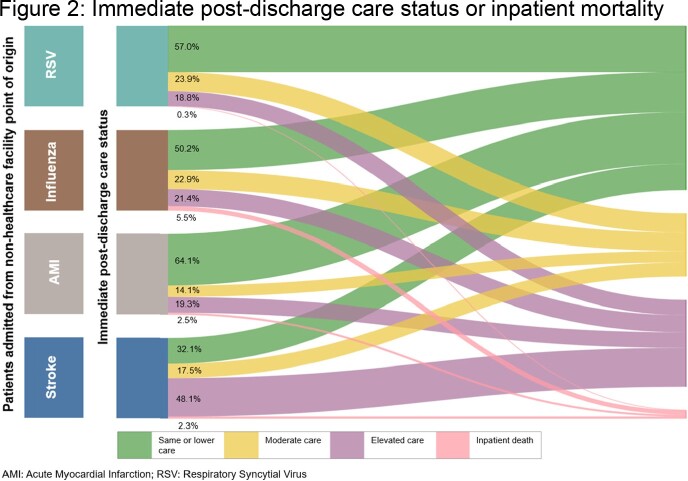

**Conclusion:**

Older adults discharged from RSV hospitalizations require considerable care with needs comparable to influenza and AMI discharges. Given the substantial care requirements of RSV hospitalizations in older adults, focus on disease prevention efforts for RSV are needed.

**Disclosures:**

**Reiko Sato, PhD**, Pfizer Inc: employee|Pfizer Inc: Stocks/Bonds **Jennifer Judy, MS, PhD**, Pfizer: Stocks/Bonds **Kari Yacisin, M.D.**, Pfizer: Employee|Pfizer: Stocks/Bonds **Elizabeth Begier, M.D., M.P.H.**, Pfizer: EB is an employee of Pfizer, the sponsor of this study|Pfizer: Stocks/Bonds **Poorva Sardana, MSc**, Pfizer Inc: Contracted Research **Neha Agrawal, MA**, Pfizer Inc: Contracted Research **Anchita Goswami, MSc**, Pfizer Inc: Contracted Research **Manvi Sharma, RPh, MBA, PhD**, Pfizer Inc: Contracted Research

